# Rib Reverberation: An important New Artifact in Lung Ultrasound.

**DOI:** 10.24908/pocusj.v10i01.17768

**Published:** 2025-04-15

**Authors:** Yulei Cao, Jennifer K. Sun, Cameron M. Baston

**Affiliations:** 1 Department of Medicine, Hospital of the University of Pennsylvania, Philadelphia, PA, USA; 2 Department of Pediatrics, Children's Hospital of Philadelphia, Philadelphia, PA, USA

**Keywords:** lung ultrasound, artifact, A-line, rib ultrasound, reverberation artifact

## Abstract

Point of care ultrasound of the lungs has emerged as a crucial tool in the evaluation of hypoxemia in critical care and hospital medicine settings. The ability to distinguish the horizontal A-line artifact from other lung pathology is essential for guiding clinical decision making. Typically, ribs and their acoustic shadows are used as anchoring anatomy to ensure visualization of pleura and parenchyma. We present a case of horizontal reverberation artifacts incidentally observed over a rib during a point of care lung ultrasound (LUS) in a 77-year-old patient with persistent hypoxic respiratory failure. Describing these reverberation artifacts caused by ribs is important to decrease the chance of misinterpretation.

## Introduction

**Figure 1.**
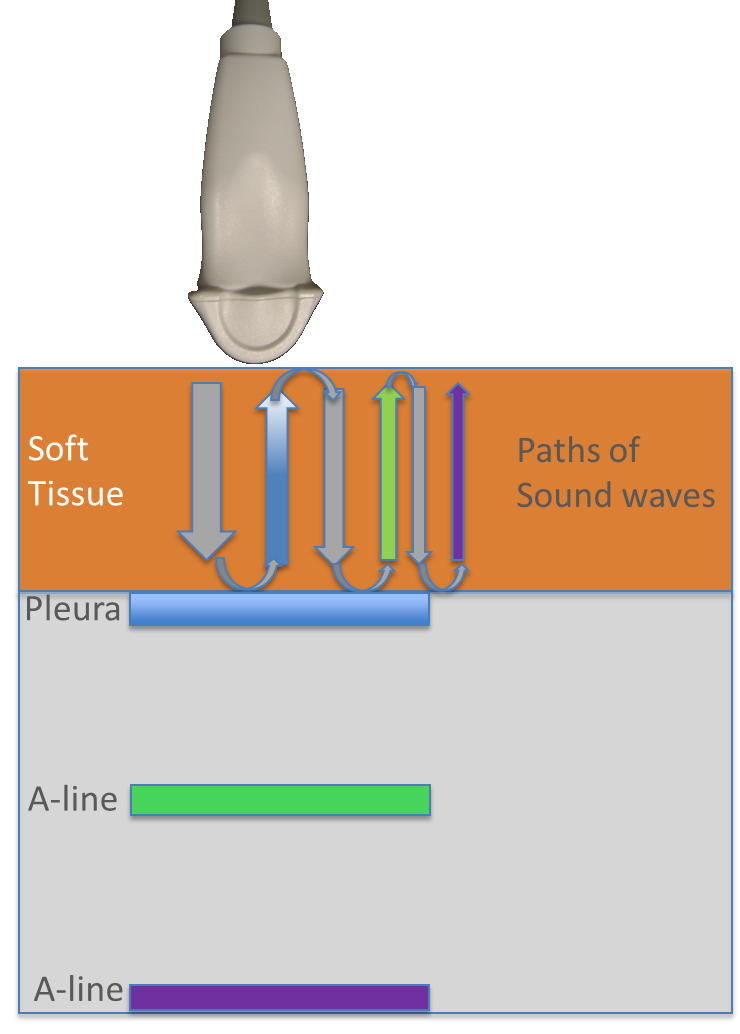
Schematic of the path of color-coded sound waves and the reverberation artifacts known as A-lines.

Point of care lung ultrasound (LUS) has been gaining an increasing amount of interest and attention as an effective and feasible tool to evaluate various pulmonary pathologies [1]. Often described as a study of artifacts, LUS takes advantage of the unique sonographic characteristics of the thorax. The artifacts generated by the pleural-parenchymal interface have been shown to be highly reliable under different pathological states, correlating more closely with computed tomography (CT) findings than chest radiograph [2]. Horizontal reverberating artifacts, also known as A-lines, are repetitive, horizontal, echogenic lines generated by sound waves reverberating between the air-filled lung/pleural interface and the transducer (Figure 1). These artifacts are present in other clinical scenarios such as dilated bowel or pneumoperitoneum but are used in LUS to identify air under the pleura. The presence or absence of lung sliding can help determine whether the patient has well-aerated lung or pneumothorax.

**Figure 2.**
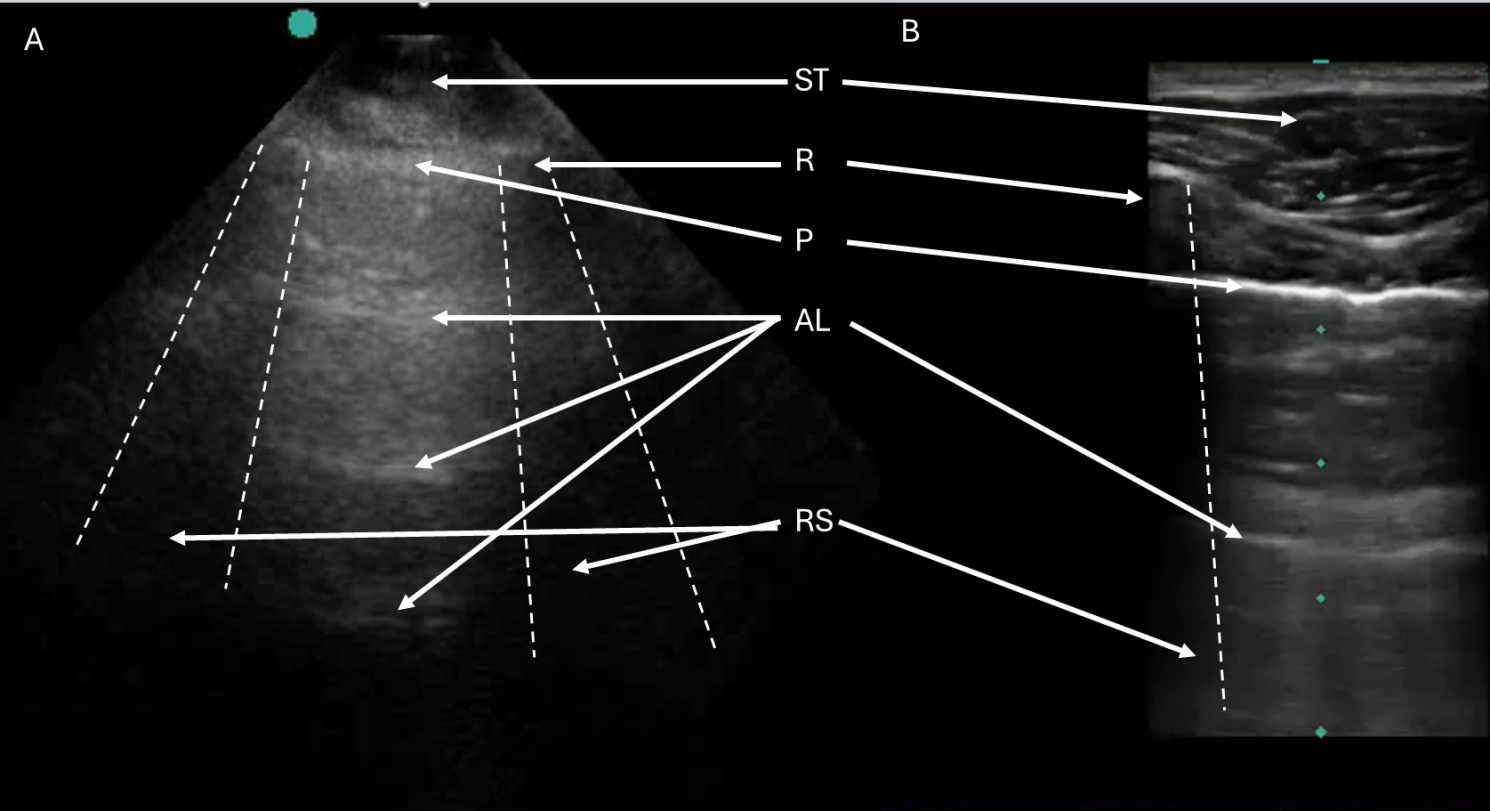
Panel A shows labeled anchoring anatomy of normal lungs including soft tissue (ST), ribs (R), Rib shadows (RS), Pleura (P), and A-lines (AL). Panel A shows an image acquired with a phased array transducer. Panel B shows an image acquired with a linear transducer.

In LUS, ribs and rib shadows are helpful in identifying the correct probe positioning, as the upper and lower ribs and their acoustic shadows provide anchoring anatomy. Typically, the ultrasound probe would be positioned longitudinally, perpendicular to the ribs, with the upper and lower ribs projecting acoustic shadows on the two edges of the ultrasound field and the lung tissues in between – sometimes known as the “batwing sign” (Figure 2)[3]. LUS can, however, also be performed oblique to the ribs to access a longer portion of pleura per transducer [3, 4]. In either orientation, the pleura is deep to the ribs and A-line artifacts appear at equidistantly spaced depths below that. This serves as a clear contrast to the vertical B-line artifacts that appear reliably in the alveolar-interstitial syndrome or the hepatized appearance of consolidated lung [5, 6].

False negatives in LUS typically occur when there are bullae or a layer of well-aerated alveoli in between pathology and the pleural surface. This prevents the sound waves from penetrating deeper and visualizing anything beyond that first layer. Differentiating A-lines from rib shadow is easy, and not typically a source of error. Here, however, we present a case report where we incidentally observed repeating horizontal hyperechoic artifacts deep to a rib, posing a potential challenge on the interpretation of LUS for novices.

## Case report

**Figure 3.**
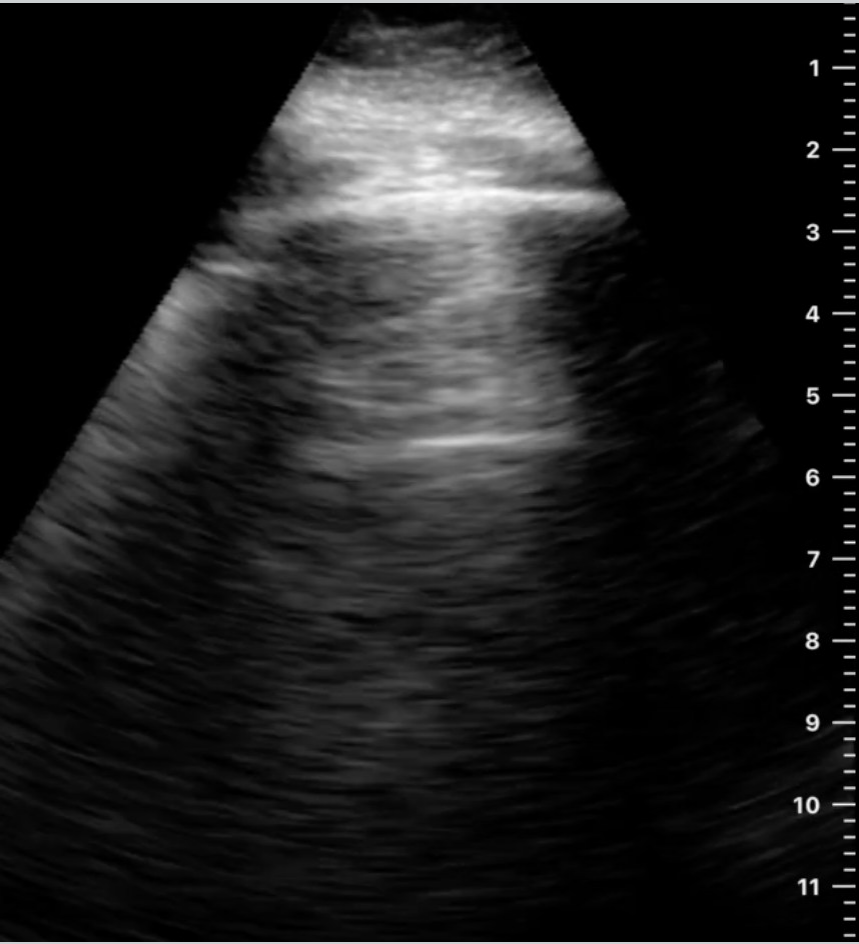
The horizontal reverberation artifact visualized below a rib in left posterolateral zone in a 77-year-old woman.

**Figure 4.**
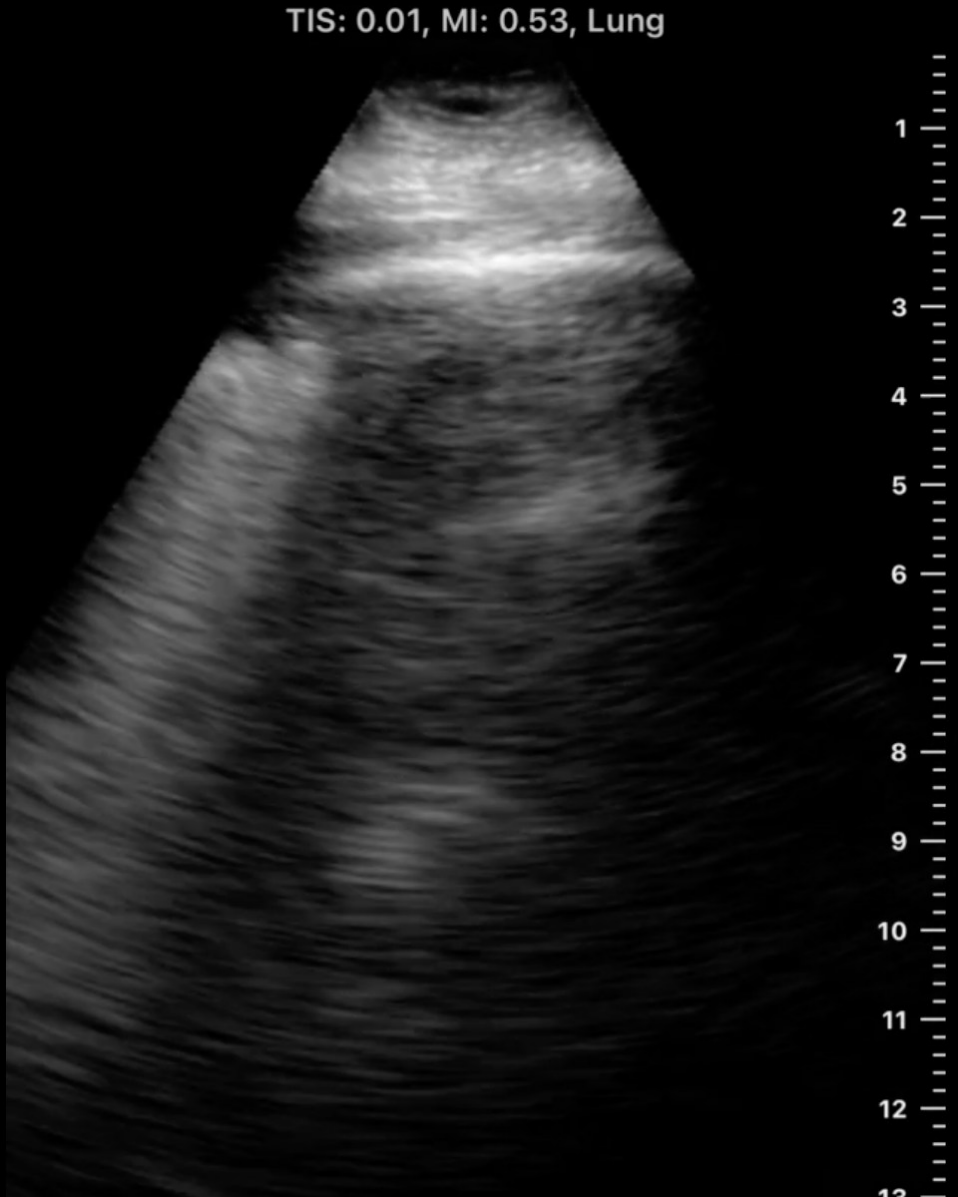
Confluent B-line artifacts on the left in lung parenchyma while horizontal reverberation artifact visualized on the right under a rib.

A 77-year-old woman with a medical history of uterine cancer on maintenance trastuzumab and recently initiated pembrolizumab (3 weeks prior to presentation), was admitted to our tertiary academic hospital for progressive dyspnea, hypoxia, and cough, despite outpatient antibiotic therapy. A CT scan on presentation revealed multifocal moderate to severe patchy peribronchial ground glass opacities and consolidation predominantly at the bilateral lung bases, initially thought to be consistent with multifocal bronchopneumonia. She was treated with a short course of vancomycin and cefepime. Pulmonology was consulted due to concern for immune check point pneumonitis given clinical course, timing, and negative infectious workup. The patient was initiated on methylprednisolone for grade 3 pneumonitis with improvement of symptoms but with increasing oxygen requirement. A diagnostic LUS was performed as part of the work up for persistent hypoxia using a handheld ultrasound machine (Butterfly iQ+, Butterfly, Boston MA) in the lung setting with a depth of 15 cm. The horizontal reverberation artifact can be seen in Figure 3 (Video 1) in zone 4L (left posterolateral zone). Another still image (Figure 4) shows that the appropriately aligned scan revealed consolidated B-line artifacts.

Instead of the expected hypoechoic shadows deep with respect to the rib, we observed two repetitive, horizontal, echogenic lines that were equidistant to each other. The sonographic features we observed underneath a rib could easily be misinterpreted as A-lines. Repeat CT scan was repeated on the 7^th^ day of admission which showed mild worsening of the patient's pneumonitis. The patient was monitored closely, and her oxygen requirement eventually stabilized on steroid therapy with symptom resolution. She was discharged to a skilled nursing facility with a long steroid taper. On outpatient follow-up 7 weeks after discharge, her CT showed significant decrease in peripheral and peribronchial ground glass opacites, and her hypoxia had resolved.

**Figure 5.**
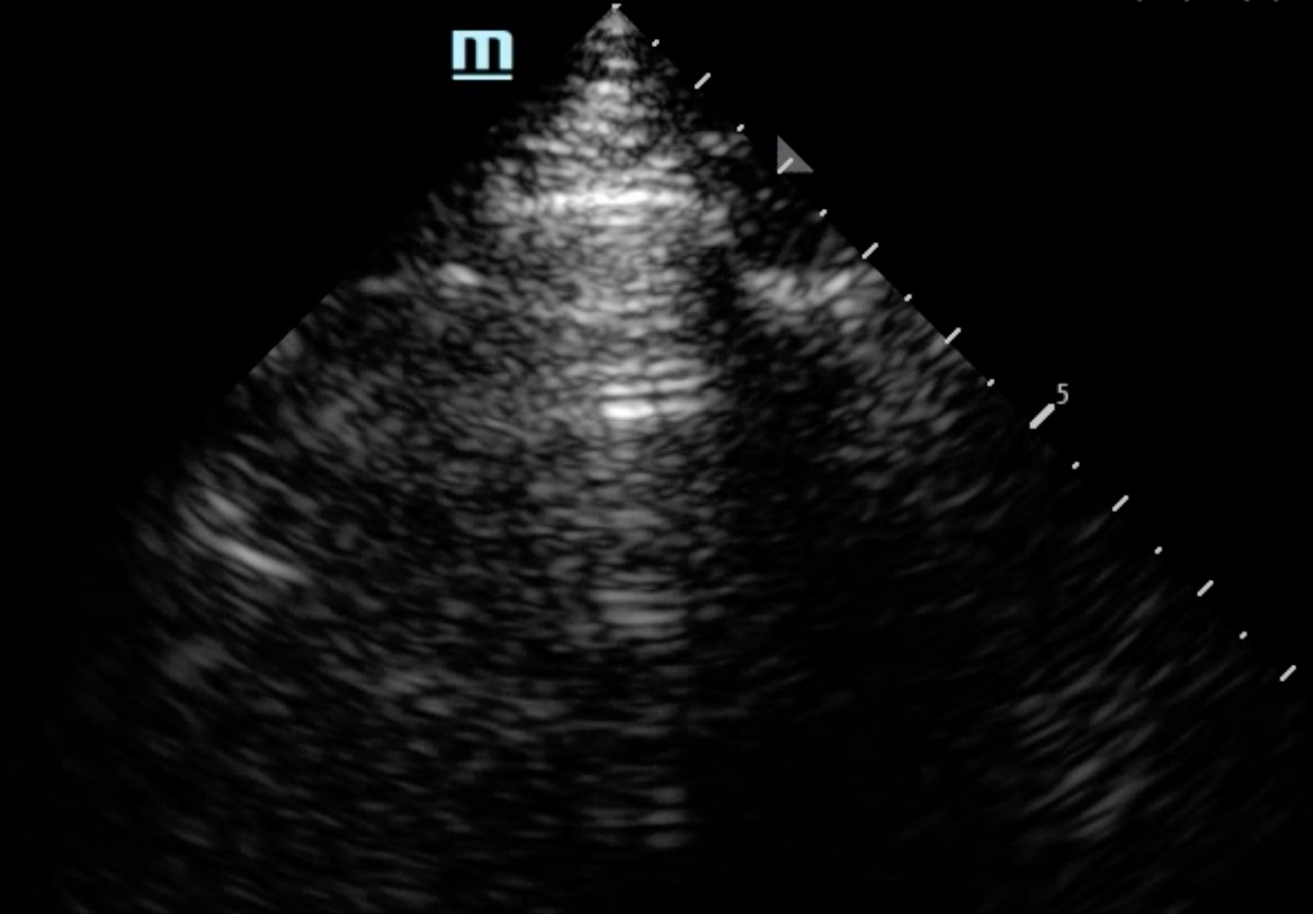
The horizontal reverberation artifact under a rib observed with a cart-based machine

**Figure 6.**
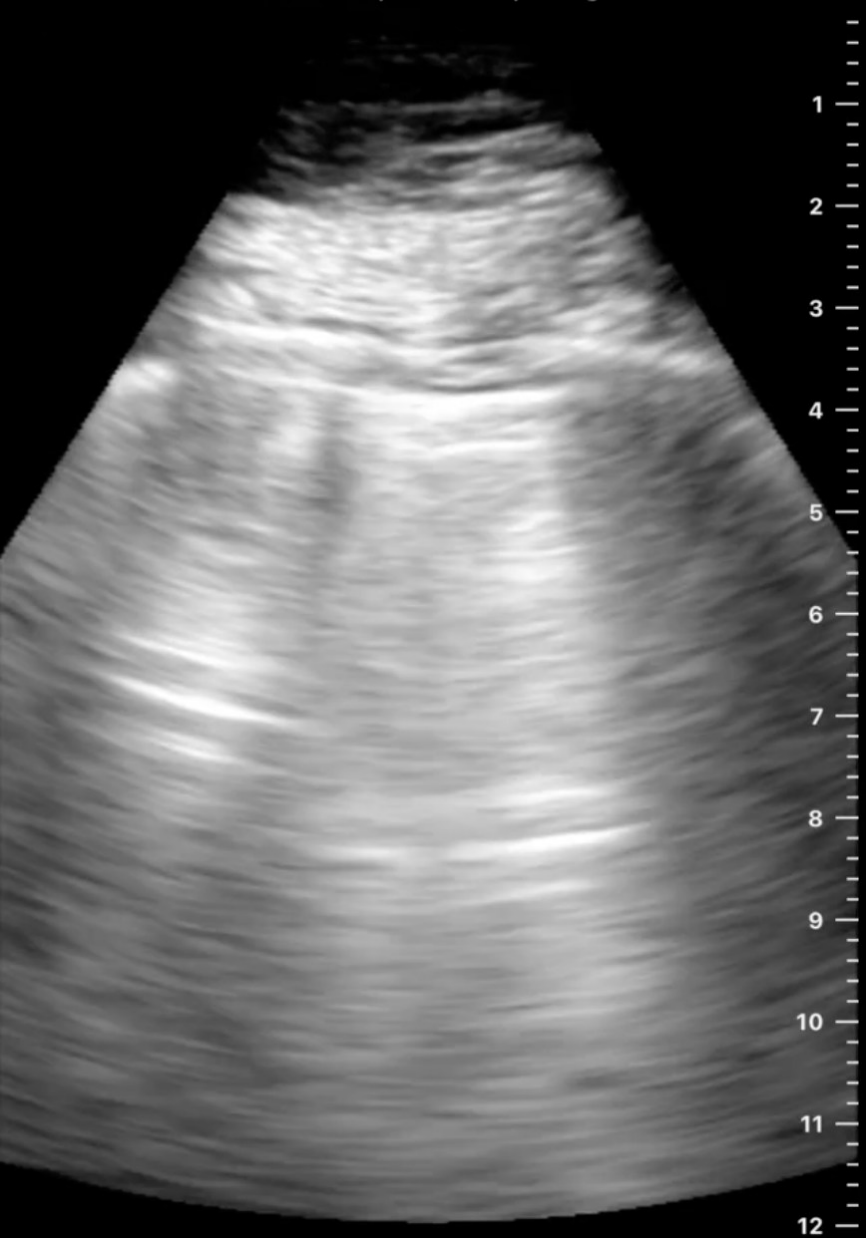
Horizontal reverberation artifact under a rib with A-line in lung parenchyma in a side-to-side view.

We determined that this finding is not necessarily specific to one machine or preset. In investigating this artifact, it was also seen from a cart-based machine (Mindray, NJ, USA) (Figure 5, Video 2), and this example in another setting showing rib reverberating artifact next to lung parenchymal A-line (Figure 6, Video 3).

## Discussion

We present a case report describing horizontal sonographic artifacts that resemble A-lines generated by a rib. Rib ultrasound has been studied typically for the identification of fractures [7]. Typically, since high density bone blocks ultrasound waves from traveling down, ribs appear with a hyperechoic horizontal line at the interface of the soft tissue and the cortical bone, with acoustic shadow continuing down the rest of the ultrasound field [8].

Our case report highlights that the rib artifact can resemble classic A-line artifact, with regularly spaced hyperechoic horizontal lines. In-between these horizontal artifacts, the background of hypoechoic field also resembles lung parenchyma. While the exact mechanism is unclear, we suspect that the change in acoustic impedance across cortical bone and soft tissue serves as a strong reflective surface, causing the reverberation artifacts between the transducer and the periosteum, analogous to that seen between the pleura and the transducer in A-lines. Certain conditions that change bone configurations further, such as osteoporosis, may increase the chance of this artifact being observed. As machine settings attempt to maximize lung artifacts, this finding may become more common. This is important as these rib artifacts could be misinterpreted as aerated lung, especially in a patient where habitus makes identification of anatomy more challenging. In addition, the lack of “sliding” artifact would potentially contribute to additional diagnostic errors if it were misinterpreted as a marker of pneumothorax or pleurodesis. In the images that we obtained, true lung tissue was identified based on the sliding motion that occurs with respiratory cycles compared to rib A-line artifacts, which are static. It highlights the importance for learners to start LUS by palpating rib edges on patients, positioning the prob longitudinally, perpendicular to the ribs, with the upper and lower ribs visualized on left and right side of the screen, and identifying lung parenchyma by the sliding motion.

We conducted PubMed and Embase search for articles from 1975 to now, with keywords including reverberation, ultrasound, bone, periosteum, or ribs (supplement A). We did not find clear documentation of long path horizontal reverberation artifacts under the ribs that closely resemble A-lines. One close representation is “M line.” This short path reverberation artifact observed within a rib shadow is briefly described by Dr. Lichtenstein, one of the pioneers of LUS, reportedly named for one of his colleagues, Dr. Macone [9]. The prior description does not elaborate on the potential clinical pitfalls that this artifact can lead to in the era of more widespread lung ultrasonography. We also noted that rib reverberation artifact was present in one picture of normal LUS but without specific notation [8], but without description in the text or image. Other than that, while there are descriptions of this sort of horizontal artifact resulting from fetal craniums, we could not find it mentioned elsewhere in the literature[10, 11]. It is important to highlight the educational value of our observation as these rib reverberation artifacts are not widely discussed and can easily be mistaken as A-lines that represent lung parenchyma, leading to diagnostic errors for learners.

LUS is described as simple to perform, with as few as 17 scans being associated with assessments for proficiency [12]. As the technology becomes more widely used, however, it becomes ever more essential to understand potential pitfalls. We report this case describing horizontal artifacts arising from the rib surface that closely resemble A-lines as an important finding to be aware of when interpreting LUS. In the future, it would be useful to identify clinical features or machine settings that are associated with this finding. For now, it is important that practitioners are aware of the potential for misinterpretation, and the importance (as always) of highlighting the anchoring anatomy of the ribs in the longitudinal plane to avoid mistaking rib periosteum for pleura.








