# Supplementary material for: Rib Reverberation: An important New Artifact in Lung Ultrasound
Source: POCUS J. 2025 Apr 15;10(1):99–103. doi: 10.24908/pocusj.v10i01.17768 (PMC12057472; doi:10.24908/pocusj.v10i01.17768)
Supplement: Supplementary file 1 [file pocusj-10-01-17768-s001.pdf]

Supplement A. Pubmed and Embase literature search code and results.

PubMed search
#1: "reverberation*"[Title/Abstract]
#2: ("ultrasound*"[Title/Abstract] OR "ultrason*"[Title/Abstract] OR "echograph*"[Title/Abstract] OR "Ultrasonography"[MeSH Terms])
#3: ("rib"[Title/Abstract] OR "Ribs"[Title/Abstract] OR "Ribs"[MeSH Terms] OR "Periosteum"[Title/Abstract] OR "Periosteum"[MeSH Terms] OR "Bone and Bones"[MeSH Terms] OR "bone"[Title/Abstract] OR "bones"[Title/Abstract])
#4: #1 AND #2 AND #3

30 results as shown below

1: Soulioti DE, Espindola D, Dayton PA, Pinton GF. Super-Resolution Imaging Through the Human Skull. IEEE Trans Ultrason Ferroelectr Freq Control. 2020 Jan;67(1):25-36. doi: 10.1109/TUFFC.2019.2937733. Epub 2019 Sep 4. PMID: 31494546.

2: Fink M, Montaldo G, Tanter M. Time-reversal acoustics in biomedical engineering. Annu Rev Biomed Eng. 2003;5:465-97. doi: 10.1146/annurev.bioeng.5.040202.121630. PMID: 14527319.

3: Pan YC, Chan HL, Kong X, Hadjiiski LM, Kripfgans OD. Multi-class deep learning segmentation and automated measurements in periodontal sonograms of a porcine model. Dentomaxillofac Radiol. 2022 Mar 1;51(3):20210363. doi: 10.1259/dmfr.20210363. Epub 2021 Nov 23. PMID: 34762512; PMCID: PMC8925874.

4: Ostras O, Soulioti DE, Pinton G. Diagnostic ultrasound imaging of the lung: A simulation approach based on propagation and reverberation in the human body. J Acoust Soc Am. 2021 Nov;150(5):3904. doi: 10.1121/10.0007273. PMID: 34852581.

5: Zhuang B, Rohling R, Abolmaesumi P. Region-of-Interest-Based Closed-Loop Beamforming for Spinal Ultrasound Imaging. IEEE Trans Ultrason Ferroelectr Freq Control. 2019 Aug;66(8):1266-1280. doi: 10.1109/TUFFC.2019.2914957. Epub 2019 May 6. PMID: 31059437.

6: Bruce M, Kolokythas O, Ferraioli G, Filice C, O'Donnell M. Limitations and artifacts in shear-wave elastography of the liver. Biomed Eng Lett. 2017 May 25;7(2):81-89. doi: 10.1007/s13534-017-0028-1. PMID: 30603154; PMCID: PMC6208474.

7: Mozaffarzadeh M, Minonzio C, de Jong N, Verweij MD, Hemm S, Daeichin V. Lamb Waves and Adaptive Beamforming for Aberration Correction in Medical Ultrasound Imaging. IEEE Trans Ultrason Ferroelectr Freq Control. 2021 Jan;68(1):84-91. doi: 10.1109/TUFFC.2020.3007345. Epub 2020 Dec 23. PMID: 32746204.

8: Maeda M, Maeda N, Masuda K, Nagano T, Tanaka Y. Diagnosis of Lumbar Transverse Process Fractures in Orthopedic Clinics Using Sonography. J Ultrasound Med. 2022 Jul;41(7):1825-1835. doi: 10.1002/jum.15846. Epub 2021 Oct 21. PMID: 34676569.

9: Doust BD, Baum JK, Maklad NF, Doust VL. Ultrasonic evaluation of pleural opacities. Radiology. 1975 Jan;114(1):135-40. doi: 10.1148/114.1.135. PMID: 1208853.

10: Younan Y, Deffieux T, Larrat B, Fink M, Tanter M, Aubry JF. Influence of the pressure field distribution in transcranial ultrasonic neurostimulation. Med Phys. 2013 Aug;40(8):082902. doi: 10.1118/1.4812423. PMID: 23927357.

11: Hurley ME, Keye GD, Hamilton S. Is ultrasound really helpful in the detection of rib fractures? Injury. 2004 Jun;35(6):562-6. doi: 10.1016/S0020-1383(03)00263-8. PMID: 15135274.

12: Shin J, Chen Y, Malhi H, Yen JT. Ultrasonic Reverberation Clutter Suppression Using Multiphase Apodization With Cross Correlation. IEEE Trans Ultrason Ferroelectr Freq Control. 2016 Nov;63(11):1947-1956. doi: 10.1109/TUFFC.2016.2597124. PMID: 27824570; PMCID: PMC5135291.

13: Levitt RG, Geisse GG, Sagel SS, Stanley RJ, Evens RG, Koehler RE, Jost RG. Complementary use of ultrasound and computed tomography in studies of the pancreas and kidney. Radiology. 1978 Jan;126(1):149-52. doi: 10.1148/126.1.149. PMID: 619398.

14: Fatemi A, Berg EAR, Rodriguez-Molares A. Studying the Origin of Reverberation Clutter in Echocardiography: In Vitro Experiments and In Vivo Demonstrations. Ultrasound Med Biol. 2019 Jul;45(7):1799-1813. doi: 10.1016/j.ultrasmedbio.2019.01.010. Epub 2019 Apr 30. PMID: 31053427.

15: Hacihaliloglu I, Guy P, Hodgson AJ, Abugharbieh R. Automatic extraction of bone surfaces from 3D ultrasound images in orthopaedic trauma cases. Int J Comput Assist Radiol Surg. 2015 Aug;10(8):1279-87. doi: 10.1007/s11548-014-1141-6. Epub 2015 Jan 1. PMID: 25549799.

16: Brinker ST, Balchandani P, Seifert AC, Kim HJ, Yoon K. Feasibility of Upper Cranial Nerve Sonication in Human Application via Neuronavigated Single-Element Pulsed Focused Ultrasound. Ultrasound Med Biol. 2022 Jun;48(6):1045-1057. doi: 10.1016/j.ultrasmedbio.2022.01.022. Epub 2022 Mar 25. PMID: 35341621.

17: Becher H, Tiemann K. Verbesserung der Endokarderkennung mittels modifizierter transthorakaler Echokardiographie unter Verwendung der zweiten harmonischen Schwingungen (Tissue Harmonic Imaging) [Improved endocardium imaging using modified transthoracic echocardiography with the second harmonic

frequency (tissue harmonic imaging)]. Herz. 1998 Dec;23(8):467-73. German. doi: 10.1007/BF03043753. PMID: 10023580.

18: Zhuang B, Rohling R, Abolmaesumi P. Accumulated Angle Factor-Based Beamforming to Improve the Visualization of Spinal Structures in Ultrasound Images. IEEE Trans Ultrason Ferroelectr Freq Control. 2018 Feb;65(2):210-222. doi: 10.1109/TUFFC.2017.2781726. PMID: 29389653.

19: Jakovljevic M, Bottenus N, Kuo L, Kumar S, Dahl JJ, Trahey GE. Blocked Elements in 1-D and 2-D Arrays-Part II: Compensation Methods as Applied to Large Coherent Apertures. IEEE Trans Ultrason Ferroelectr Freq Control. 2017 Jun;64(6):922-936. doi: 10.1109/TUFFC.2017.2683562. Epub 2017 Mar 16. PMID: 28328505; PMCID: PMC5834306.

20: Govinahallisathyanarayana S, Ning B, Cao R, Hu S, Hossack JA. Dictionary learning-based reverberation removal enables depth-resolved photoacoustic microscopy of cortical microvasculature in the mouse brain. Sci Rep. 2018 Jan 17;8(1):985. doi: 10.1038/s41598-017-18860-3. PMID: 29343801; PMCID: PMC5772684.

21: Enzmann DR, Britt RH, Lyons B, Buxton TL, Wilson DA. Experimental study of high-resolution ultrasound imaging of hemorrhage, bone fragments, and foreign bodies in head trauma. J Neurosurg. 1981 Mar;54(3):304-9. doi: 10.3171/jns.1981.54.3.0304. PMID: 7463130.

22: Carerj S, Trono A, Zito C, Ficarra E, Luzza F, Oreto G, Arrigo F. La seconda armonica tissutale: dai principi fisici all'applicazione clinica [The second tissue harmonic signal: from physics principles to clinical application]. Ital Heart J Suppl. 2001 Oct;2(10):1078-86. Italian. PMID: 11723610.

23: Omena TP, Fontes-Pereira AJ, Costa RM, Simões RJ, von Krüger MA, Pereira WCA. Why we should care about soft tissue interfaces when applying ultrasonic diathermy: an experimental and computer simulation study. J Ther Ultrasound. 2017 Jan 27;5:3. doi: 10.1186/s40349-017-0086-y. PMID: 28149518; PMCID: PMC5270207.

24: Stanley JH, Harrell B, Horger EO 3rd. Pseudoepidural reverberation artifact: a common ultrasound artifact in fetal cranium. J Clin Ultrasound. 1986 May;14(4):251-4. doi: 10.1002/jcu.1870140404. PMID: 3084581.

25: Ahn JH, Sohn Y. Application of point-of-care ultrasound for different types of esophageal foreign bodies: three case reports: A CARE-compliant article. Medicine (Baltimore). 2020 Jan;99(4):e18893. doi: 10.1097/MD.0000000000018893. PMID: 31977900; PMCID: PMC7004603.

26: Chadha D, Kedar RP, Malde HM. Sonographic detection of pneumoperitoneum: an experimental and clinical study. Australas Radiol. 1993 May;37(2):182-5. doi:

10.1111/j.1440-1673.1993.tb00046.x. PMID: 8512509.

27: Wear KA. A method for improved standardization of in vivo calcaneal time-domain speed-of-sound measurements. IEEE Trans Ultrason Ferroelectr Freq Control. 2008 Jul;55(7):1473-9. doi: 10.1109/TUFFC.2008.822. PMID: 18986936; PMCID: PMC9148199.

28: Scherer U, Rochels R. Artefakte im A- und B-Bild-Echogramm nach Implantation von Kunstlinsen [Artifacts in A and B-scan echograms following the implantation of artificial lenses]. Ophthalmologica. 1983;187(4):192-5. German. doi: 10.1159/000309325. PMID: 6657181.

29: De Flaviis L, Scaglione P, Del Bò P, Nessi R. Detection of foreign bodies in soft tissues: experimental comparison of ultrasonography and xeroradiography. J Trauma. 1988 Mar;28(3):400-4. doi: 10.1097/00005373-198803000-00018. PMID: 3280817.

30: Schoenecker SA, Pretorius DH, Manco-Johnson ML. Artifacts seen commonly on ultrasonography of the fetal cranium. J Reprod Med. 1985 Jul;30(7):541-4. PMID: 4032391.

Embase search
#1: 'reverberation':ti,ab
#2: 'ultrasound'/exp OR 'ultrasound*':ti,ab OR 'ultrason*':ti,ab OR 'echograph*':ti,ab
#3: 'rib'/exp OR 'periosteum'/exp OR 'bone'/exp OR 'rib':ti,ab OR 'Ribs':ti,ab OR 'Periosteum':ti,ab OR 'bone':ti,ab OR 'bones':ti,ab
#4: #1 AND #2 AND #3

35 results as shown below

1: Prajapati R.; Desai P.; Patel C.; Kabrawala M. Linear endoscopic ultrasound imaging of aberrant right subclavian artery (arteria lusoria) Gut (2023) 72 Supplement 1 (A216-A217 DOI: 10.1136/gutjnl-2023-IDDF.211

2: Brinker S.T.; Balchandani P.; Seifert A.C.; Kim H.-J.; Yoon K. Feasibility of Upper Cranial Nerve Sonication in Human Application via Neuronavigated Single-Element Pulsed Focused Ultrasound. Ultrasound in Medicine and Biology (2022) 48:6 (1045-1057). DOI: 10.1016/j.ultrasmedbio.2022.01.022

3: Pan Y.-C.; Chan H.-L.; Kong X.; Hadjiiski L.M.; Kripfgans O.D. Multi-class deep learning segmentation and automated measurements in periodontal sonograms of a porcine model. Dento maxillo facial radiology (2022) 51:3 (20210363). DOI: 10.1259/dmfr.20210363

4: Vasilieva Y.P.; Skripchenko N.V.; Klimkin A.V.; Bedova M.A.; Levina O.A. Novel approach to comprehensive diagnosis of intracranial hypertension in children with neuroinfections. Voprosy Prakticheskoi Pediatrii (2022) 17:5 (90-100). DOI: 10.20953/1817-7646-2022-5-90-100

5: Wang Y.-H.; Yang C.-S.; Chang K.-C.; Chang S.-L.; Cheng C.-Y.; Huang Y.-L. The applications of real-time imaging with transillumination, ultrasound, and Doppler for thread lifting. Dermatologica Sinica (2022) 40:1 (44-47). DOI: 10.4103/ds.ds_1_22

6: Ostras O.; Soulioti D.E.; Pinton G. Diagnostic ultrasound imaging of the lung: A simulation approach based on propagation and reverberation in the human body. The Journal of the Acoustical Society of America (2021) 150:5 (3904). DOI: 10.1121/10.0007273

7: Lee J.; Kang H.-J.; Yoon J.H.; Lee J.M. Ultrasound-guided transient elastography and two-dimensional shear wave elastography for assessment of liver fibrosis: Emphasis on technical success and reliable measurements. Ultrasonography (2021) 40:2 (217-227). DOI: 10.14366/usg.20036

8: Mozaffarzadeh M.; Minonzio C.; de Jong N.; Verweij M.D.; Hemm S.; Daeichin V. Lamb Waves and Adaptive Beamforming for Aberration Correction in Medical Ultrasound Imaging. IEEE transactions on ultrasonics, ferroelectrics, and frequency control (2021) 68:1 (84-91). DOI: 10.1109/TUFFC.2020.3007345

9: Duggan N.M.; Shokoohi H.; Liteplo A.S. The effect of depth, gain, and focus position on sonographic b-lines. Academic Emergency Medicine (2020) 27 Supplement 1 (S215-S216). DOI: 10.1111/acem.13961

10: Soulioti D.E.; Espindola D.; Dayton P.A.; Pinton G.F. Super-Resolution Imaging Through the Human Skull. IEEE transactions on ultrasonics, ferroelectrics, and frequency control (2020) 67:1 (25-36). DOI: 10.1109/TUFFC.2019.2937733

11: Ahn J.H.; Sohn Y. Application of point-of-care ultrasound for different types of esophageal foreign bodies: Three case reports: A CARE-compliant article. Medicine (United States) (2020) 99:4 Article Number: e18893. DOI: 10.1097/MD.0000000000018893

12: Zhuang B.; Rohling R.; Abolmaesumi P. Region-of-Interest-Based Closed-Loop Beamforming for Spinal Ultrasound Imaging. IEEE transactions on ultrasonics, ferroelectrics, and frequency control (2019) 66:8 (1266-1280). DOI: 10.1109/TUFFC.2019.2914957

13: Fatemi A.; Berg E.A.R.; Rodriguez-Molares A. Studying the Origin of Reverberation Clutter in Echocardiography: In Vitro Experiments and In Vivo Demonstrations. Ultrasound in Medicine and Biology (2019) 45:7 (1799-1813). DOI: 10.1016/j.ultrasmedbio.2019.01.010

14: Brescia F.; Borsatti E.; Semilia M.; Fabiani F.
Misleading artifact in vascular ultrasound - a case report. Journal of Vascular Access (2019) 20:1 (NP20). DOI: 10.1177/1129729818778929

15: Govinahallisathyanarayana S.; Ning B.; Cao R.; Hu S.; Hossack J.A. Dictionary learning-based reverberation removal enables depth-resolved photoacoustic microscopy of cortical microvasculature in the mouse brain. Scientific reports (2018) 8:1 (985). DOI: 10.1038/s41598-017-18860-3

16: Krishnan S. Artefacts in musculoskeletal ultrasound. Indian Journal of Rheumatology (2018) 13:5 Supplement 1 (S9-S16). DOI: 10.4103/0973-3698.238195

17: Makris A. Hands on clinicalworkshops 8: Airway and gastric us. Regional Anesthesia and Pain Medicine (2017) 42:5 Supplement 1 (e3). DOI: 10.1097/AAP.0000000000000656

18: Shin J.; Chen Y.; Malhi H.; Yen J.T. Ultrasonic Reverberation Clutter Suppression Using Multiphase Apodization With Cross Correlation. IEEE transactions on ultrasonics, ferroelectrics, and frequency control (2016) 63:11 (1947-1956).

19: Babaev A.; Zavlunova S.; Attubato M.; Martinsen B.; Mintz G.; Maehara A. Orbital atherectomy plaque modification of femoropopliteal arteries: IVUS analysis and one-year outcomes from truth study. Journal of Vascular and Interventional Radiology (2016) 27:2 (e3). DOI: 10.1016/j.jvir.2016.01.014

20: Kannan S.; Tripathi R.; Mahadevan S. Reduced sensitivity to thyroid hormone: First report of mutation in thyroid hormone receptor-beta from India. Endocrine Reviews (2016) 37:2 Supplement 1. DOI: 10.1210/endo-meetings.2016.THPTA.11.SUN-320

21: Bakilan F.; Yöce G.; Biçen A.Ç.; Keten T. The place of ultrasonography in the evaluation of rib fractures. Turk Osteoporoz Dergisi (2015) 21:3 (109-112). DOI: 10.4274/tod.45477

22: Hacihaliloglu I.; Guy P.; Hodgson A.J.; Abugharbieh R. Automatic extraction of bone surfaces from 3D ultrasound images in orthopaedic trauma cases. International Journal of Computer Assisted Radiology and Surgery (2015) 10:8 (1279-1287). DOI: 10.1007/s11548-014-1141-6

23: Babaev A.; Zavlunova S.; Attubato M.; Maehara A. Tissue removal assessment with ultrasound of the SFA and popliteal study (TRUTH): Orbital atherectomy acute data and intravascular ultrasound analysis. Journal of the American College of Cardiology (2014) 64:11 SUPPL. 1 (B40).

24: Reusz G.; Sarkany P.; Gal J.; Csomos A. Needle-related ultrasound artifacts and their importance in anaesthetic practice. British Journal of Anaesthesia (2014) 112:5 (794-802). DOI: 10.1093/bja/aet585

25: Babakhanian M.; Nowroozi B.; Saddik G.; Maccabi A.; Bajwa N.; Grundfest W. Acoustic characterization of low intensity focused ultrasound system through skull. Journal of Therapeutic Ultrasound (2014) 3 Supplement 1.

26: Sermeus L.How to start regional anesthesia with ultrasound. Regional Anesthesia and Pain Medicine (2013) 38:5 SUPPL. 1 (E98-E99). DOI: 10.1097/AAP.0b013e3182a6a39d

27: Jensen F.H.; Rocco A.; Mansur R.M.; Smith B.D.; Janik V.M.; Madsen P.T. Clicking in Shallow Rivers: Short-Range Echolocation of Irrawaddy and Ganges River Dolphins in a Shallow, Acoustically Complex Habitat. PLoS ONE (2013) 8:4 Article Number: e59284. DOI: 10.1371/journal.pone.0059284

28: Nalos M.; Kot M.; McLean A.S.; Lichtenstein D. Bedside lung ultrasound in the care of the critically ill. Current Respiratory Medicine Reviews (2010) 6:4 (271-278). DOI: 10.2174/157339810793563767

29: Wear K.A. A method for improved standardization of in vivo calcaneal time-domain speed-of-sound measurements. IEEE transactions on ultrasonics, ferroelectrics, and frequency control (2008) 55:7 (1473-1479).

30: Hurley M.E.; Keye G.D.; Hamilton S. Is ultrasound really helpful in the detection of rib fractures? Injury (2004) 35:6 (562-566). DOI: 10.1016/S0020-1383(03)00263-8

31: De Flaviis L.; Scaglione P.; Del Bo P.; Nessi R. Detection of foreign bodies in soft tissues: Experimental comparison of ultrasonography and xeroradiography. Journal of Trauma (1988) 28:3 (400-404).

32: Schoenecker S.A.; Pretorius D.H.; Manco-Johnson M.L. Artifacts seen commonly on ultrasonography of the fetal cranium. Journal of Reproductive Medicine for the Obstetrician and Gynecologist (1985) 30:7 (541-544). Date of Publication: 1985

33: Masuzawa H.; Sato J.; Kamitani H. Electronic sector-scanning ultrasonography for diagnosis and guidance in surgery of brain and spinal cord. Neurologia Medico-Chirurgica (1984) 24:8 (545-556).

34: Enzmann D.R.; Britt R.H.; Lyons B. Experimental study of high-resolution ultrasound imaging of hemorrhage, bone fragments, and foreign bodies in head trauma. Journal of Neurosurgery (1981) 54:3 (304-309). DOI: 10.3171/jns.1981.54.3.0304

35: Doust B.D.; Baum J.K.; Maklad N.F.; Doust V.L. Ultrasonic evaluation of pleural opacities. Radiology (1975) 114:1 (135-140). DOI: 10.1148/114.1.135
